# Racial and Ethnic Disparities in the Trends and Outcomes of Cardiogenic Shock Complicating Peripartum Cardiomyopathy

**DOI:** 10.1001/jamanetworkopen.2022.20937

**Published:** 2022-07-05

**Authors:** Titilope Olanipekun, Temidayo Abe, Valery Effoe, Obiora Egbuche, Paul Mather, Melvin Echols, Demilade Adedinsewo

**Affiliations:** 1 Department of Hospital Medicine, Covenant Health System, Knoxville, Tennessee; 2 Department of Internal Medicine, Morehouse School of Medicine, Atlanta, Georgia; 3 Department of Cardiovascular Medicine, Morehouse School of Medicine, Atlanta, Georgia; 4 Department of Interventional Cardiology, Ohio School of Medicine, Columbus; 5 Department of Cardiovascular Disease, Perelman School of Medicine, East Perelman Center for Advanced Medicine, University of Pennsylvania, Philadelphia; 6 Department of Cardiovascular Medicine, Mayo Clinic, Jacksonville, Florida

## Abstract

**Importance:**

Cardiogenic shock (CS) is a recognized complication of peripartum cardiomyopathy (PPCM) associated with poor prognosis. Although racial and ethnic disparities have been described in the occurrence and outcomes of PPCM, it is unclear if these disparities persist among patients with PPCM and CS.

**Objectives:**

To evaluate the temporal trends in CS incidence among hospitalized patients with PPCM stratified by race and ethnicity and to investigate the racial and ethnic differences in hospital mortality, mechanical circulatory support (MCS) use, and heart transplantation (HT).

**Design, Setting, and Participants:**

This multicenter retrospective cohort study included hospitalized patients with PPCM complicated by CS in the US from 2005 to 2019 identified from the National Inpatient Sample (NIS). Data analysis was conducted in November 2021.

**Exposure:**

PPCM complicated by CS.

**Main Outcomes and Measures:**

The main outcome was incidence of CS in PPCM stratified by race and ethnicity. The secondary outcome was racial and ethnic differences in hospital mortality, MCS use, and HT.

**Results:**

Of 55 804 hospitalized patients with PPCM, 1945 patients had CS, including 947 Black patients, 236 Hispanic patients, and 702 White patients, translating to an incidence rate of 35 CS events per 1000 patients with PPCM. The mean (SD) age was 31 (9) years. Black and Hispanic patients had higher CS incidence rates (39 events per 1000 patients with PPCM) compared with White patients (33 events per 1000 patients with PPCM). CS incidence rates significantly increased across all races and ethnicities over the study period. Overall, the odds of developing CS were higher in Black patients (aOR, 1.17 [95% CI, 1.15-1.57]; P < .001) and Hispanic patients (aOR, 1.37 [95% CI, 1.17-1.59]; P < 001) compared with White patients during the study period. Compared with White patients, the odds of in-hospital mortality were higher in Black (adjusted odds ratio [aOR], 1.67 [95% CI, 1.21-2.32]; *P* = .002) and Hispanic (aOR, 2.20 [95% CI, 1.45-3.33]; *P* < .001) patients. Hispanic patients were more likely to receive any type of MCS device (aOR, 2.23 [95% CI, 1.60-3.09]; *P* < .001), intraaortic balloon pump (aOR, 1.65 [95% CI, 1.11-2.44]; *P* < .001), and ventricular assisted device (aOR, 4.45 [95% CI, 2.45-8.08]; *P* < .001), compared with White patients. Black patients were more likely to receive VAD (aOR, 2.69 [95% CI, 1.63-4.42]; *P* < .001) compared with White patients. Black and Hispanic patients were significantly less likely to receive HT compared with White patients (Black patients: aOR, 0.51 [95% CI, 0.33-0.78]; *P* = .02; Hispanic patients: aOR, 0.15 [95% CI, 0.06-0.42]; *P* < .001).

**Conclusions and Relevance:**

These findings highlight significant racial disparities in mortality and HT among hospitalized patients with PPCM complicated by CS in the US. More research to identify factors of racial and ethnic disparities is needed to guide interventions to improve outcomes of patients with PPCM.

## Key Points

**Question:**

Are there racial or ethnic differences in the incidence of cardiogenic shock (CS) and associated in-hospital outcomes among hospitalized patients with peripartum cardiomyopathy (PPCM) in the US?

**Findings:**

In this cohort study of 55 804 hospitalized patients with PPCM complicated by CS, Black and Hispanic patients had the highest CS incidence compared with White patients during the study period. Despite higher mechanical circulatory support use, Black and Hispanic patients had higher odds of inpatient mortality, compared with White patients.

**Meaning:**

These findings suggest that more research to identify factors of racial and ethnic disparities in patient outcomes is needed to guide interventions targeted at improving outcomes in patients with PPCM.

## Teaser

This cohort study evaluates temporal trends and racial and ethnic disparities in cardiogenic shock incidence and outcomes among hospitalized patients with peripartum cardiomyopathy.

## Introduction

*Peripartum cardiomyopathy* (PPCM) is defined as heart failure secondary to left ventricular systolic dysfunction recognized 1 month prior to childbirth or in the 5 months following childbirth without another identifiable cause in a patient with no known previous structural heart disease.^1^ In the US, the incidence of PPCM ranges from 1 in 1000 to 1 in 4000 live births and likely is increasing.^1,2,3^

The incidence of PPCM is notably higher among Black patients, accounting for more than 40% of cases.^4,5^ Statewide population studies have reported PPCM incidence rates 3- to 16-fold higher in Black patients compared with White patients.^1,4^ Additionally, Black patients have a poorer prognosis, with lower rates of left ventricular recovery, increased resource utilization, and a higher mortality risk.^4,5,6,7^

Mortality rates in PPCM range from 7% to 50%, often as a result of complications, such as cardiogenic shock (CS), thromboembolism, and arrhythmias.^4,8,9,10^ CS is reported as a complication among approximately 2% to 4% of patients with PPCM, often treated with mechanical circulatory support (MCS) and heart transplantation (HT) and is associated with poor prognosis.^2,11,12^ Although racial and ethnic disparities in PPCM incidence and all-cause mortality risks have been well described, there is limited information on clinical outcomes among patients with PPCM complicated by CS.

Using data from the National Inpatient Sample (NIS), we investigated (1) trends in the incidence of CS among hospitalized patients with PPCM stratified by race and ethnicity; and (2) racial and ethnic differences in hospital mortality, use of MCS, and HT among hospitalized patients with PPCM complicated by CS.

## Methods

For this cohort study, the requirement of an institutional review board approval and informed consent was waived by Morehouse School of Medicine because the NIS is a publicly available database with deidentified data. This study is reported following the Strengthening the Reporting of Observational Studies in Epidemiology (STROBE) reporting guideline.

### Study Design

Data were sourced from the NIS database, which is a subdivision of the Healthcare Cost and Utilization Project (HCUP) managed by the Agency for Healthcare Research and Quality. The NIS is the largest publicly available administrative database in the US and contains clinical data elements on inpatient diagnoses and procedures from approximately 1000 hospitals and 7 million hospitalizations each year. The NIS database represents approximately 20% of all US hospitalizations and includes the number of beds, ownerships, hospital teaching status, US region, and state.^13^ The database captures individual hospitalizations with a primary diagnosis and several secondary and procedural diagnoses, defined by *International Classification of Diseases, Ninth Revision, Clinical Modification* (*ICD-9-CM*)^14^ and *International Statistical Classification of Diseases and Related Health Problems, Tenth Revision, Clinical Modification* (*ICD-10-CM*)^15^ codes.

### Study Population, Variables, and Outcomes

Using the *ICD-9-CM* and *ICD-10-CM* codes, we identified patients hospitalized with the primary diagnosis of PPCM (*ICD-9-CM* codes 674.50, 674.51, 674.52, 674.53, and 674.54; *ICD-10-CM* code O90.3).^12,14,15^ Patients with a secondary diagnosis of CS were identified with *ICD-9-CM* code 785.51 or *ICD-10-CM* code R57.0.^14,15,16,17^ The CS administrative codes have greater than 90% positive predictive value and specificity but relatively low sensitivity (approximately 60%).^18,19^

To ensure appropriate identification of patients with CS primarily due to PPCM, we excluded patients with acute myocardial infarction during the index hospital stay using the appropriate *ICD-9-CM* and *ICD-10-CM* codes.^2,14,15^ The primary risk factors examined were race and ethnicity, categorized into 3 racial and ethnic groups: Black, Hispanic, and White. Coding for race and ethnicity in NIS combines self-reported race and ethnicity provided by the data source into 1 data element (“RACE”). If both race and ethnicity were available, ethnicity was preferred over race in setting the HCUP value for RACE.^20^ Other racial and ethnic groups (eg, American Indian, Asian or Pacific Islander) were excluded owing to small sample sizes, limiting the ability to adequately explore the study outcomes for each race and ethnicity. Sociodemographic data, including age, primary payer, household income, hospital location, teaching-hospital status, and comorbidities associated with the primary diagnosis, were extracted similar to our prior published works.^21,22,23^

The study primary outcome was in-hospital mortality among patients with PPCM complicated by CS. The secondary outcomes were MCS use and HT. We identified MCS devices: extracorporeal membrane oxygenation (ECMO), intraaortic balloon pump (IABP), and percutaneous left ventricular assist device (PLVAD) using their respective *ICD* procedure codes.^14,15^ The *ICD-9-CM* and *ICD-10-CM* diagnosis and procedural codes are available in eTable 1 in the Supplement.

### Statistical Analysis

Weighted discharge data were used for all analysis to reflect the number of PPCM and CS events in the US population, consistent with previous studies on pregnancy-related complications.^2,24^ Among patients with PPCM complicated by CS, we compared baseline comorbidities, primary payer, household income, hospital location, region, and teaching status among Black, Hispanic, and White patients. We used χ^2^ test for categorical variables and Kruskal-Wallis 1-way analysis of variance for continuous variables. Descriptive statistics were reported in frequencies and percentages for categorical variables, while continuous variables were reported using means and SDs.

Cochrane-Armitage test was used to evaluate the temporal trends in CS incidence rates among patients with PPCM, stratified by race. The rates were expressed as a percentage, or per 1000 hospitalized patients with PPCM, as appropriate. We modeled calendar year as a continuous variable using linear regression to obtain adjusted odds ratios (aORs) per year for the overall temporal trend in the incidence of CS during the study period. We also evaluated calendar year as a categorical variable, with 2005 as the reference year, to evaluate for temporal variability from year to year in CS incidence. Multivariate logistic regression was used to estimate the association between race and the primary and secondary outcomes. We also determined the association between significant patient variables and the mortality in the overall population of patients with PPCM and CS. Regression models were adjusted for age, insurance type, median household income, hospital teaching status, and significant comorbidities, including chronic kidney disease (CKD), diabetes, hypertension, obesity, hyperlipidemia, polysubstance abuse, hypothyroidism, hypertensive disorders of pregnancy, and antepartum hemorrhage (APH).

To further explore differences in the in-hospital mortality, we evaluated the odds of in-hospital mortality in Black and Hispanic patients compared with White patients among subgroups of patients categorized by age, median household income, insurance type, comorbidities (CKD, diabetes, hypothyroidism, APH, and hypertensive disorders of pregnancy), MCS use, and HT. These subgroups were specified a priori from the pathophysiological hypothesis of mortality in PPCM^25^ and previously published findings on mortality risk factors in PPCM.^26,27,28^ Each subgroup analysis was appropriately adjusted.^25^

All analyses were performed using SPSS Statistics software version 26.0 (IBM). Two-sided *P* < .05 was considered statistically significant. Data were analyzed in November 2021.

## Results

### Baseline Characteristics

**Figure 1.**
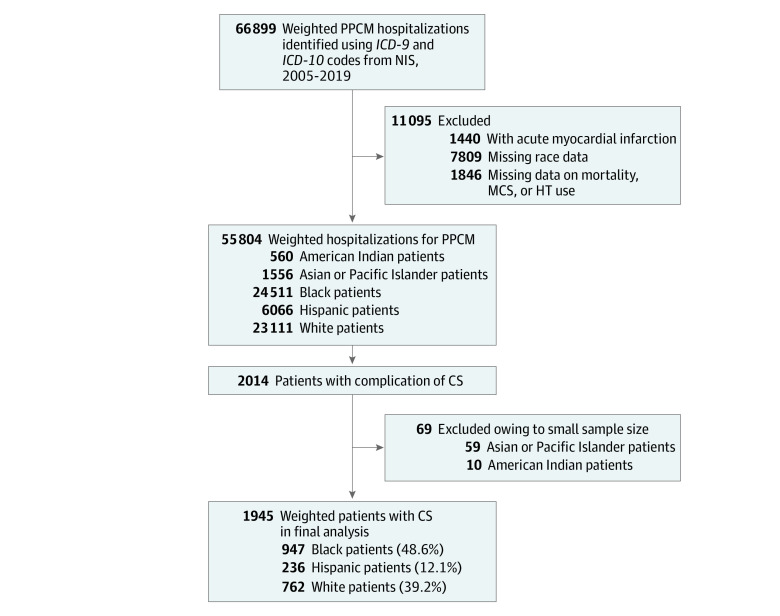
Study Recruitment Flowchart CS indicates cardiogenic shock; HT, heart transplant; *ICD-9*, *International Classification of Diseases, Ninth Revision*; *ICD-10*, *International Statistical Classification of Diseases and Related Health Problems, Tenth Revision;* MCS, mechanical circulatory support; NIS, National Inpatient Sample; PPCM, peripartum cardiomyopathy.

During the study period of 2005 to 2019, 55 804 hospitalized patients with PPCM were identified, of whom 24 511 were Black (43.9%), 6066 were Hispanic (10.9%), and 23 111 were White (41.4%). A total of 2014 patients had a complication of CS, and after excluding 10 American Indian patients and 59 Asian or Pacific Islander patients, 1945 patients met the inclusion criteria, representing our final study population (Figure 1). Among these patients, 947 (48.6%) were Black, 236 (12.1%) were Hispanic, and 762 (39.2%) were White (Table 1).

**Table 1.** Baseline Characteristics of Hospitalizations With Peripartum Cardiomyopathy and Cardiogenic Shock Stratified by Race and Ethnicity

Variables	Patients, No. (%)			*P* value
	Black (n = 947)	Hispanic (n = 236)	White (n = 762)	
Age, mean (SD), y	32 (8)	31 (9)	32 (9)	.06
Primary payer				
Medicare	201 (21.2)	15 (6.4)	52 (6.8)	<.001
Medicaid	519 (54.8)	119 (50.4)	351 (46.1)	
Private	192 (20.3)	73 (30.9)	315 (41.3)	
Self-pay	20 (2.1)	15 (6.4)	10 (1.3)	
Others	15 (1.6)	14 (5.9)	34 (4.5)	
Quartile of median household income^a^				
0-25th	467 (49.3)	64 (27.1)	185 (24.3)	<.001
26th-50th	181 (19.1)	65 (27.5)	226 (29.6)	
51st-75th	189 (20.0)	64 (27.1)	208 (27.3)	
75th-100th	110 (11.6)	43 (18.2)	143 (18.8)	
Hospital teaching status and location				
Rural	9 (1.0)	5 (2.1)	29 (3.8)	<.001
Urban				
Nonteaching	67 (7.1)	33 (14.0)	107 (14.0)	
Teaching	870 (91.9)	198 (83.8)	626 (82.2)	
Hospital bed size^a^				
Small	14 (1.5)	0	25 (3.3)	<.001
Medium	111 (11.7)	39 (16.5)	126 (16.6)	
Large	822 (86.8)	197 (83.5)	610 (80.1)	
Comorbidities				
Hypothyroidism	54 (5.7)	20 (8.5)	54 (7.1)	.28
Obesity	218 (23)	58 (21.2)	95 (12.5)	<.001
Diabetes mellitus	118 (12.5)	25 (10.6)	40 (5.2)	<.001
Chronic kidney disease	248 (26.2)	34 (14.4)	90 (11.8)	<.001
History of chronic hypertension	391 (41.3)	40 (16.9)	174 (22.9)	<.001
Polysubstance use^b^	80 (8.4)	5 (2.1)	80 (10.5)	<.001
Hyperlipidemia	80 (8.4)	5 (2.1)	50 (6.6)	.01
Hypertensive disorder of pregnancy^c^	34 (3.6)	13 (5.3)	21 (2.8)	<.001
Antepartum hemorrhage	9 (1.0)	10 (4.2)	5 (0.7)	<.001

^a^ Estimated median household incomes are zip code–specific, updated annually and classified into 4 quartiles indicating the poorest to wealthiest populations. Bed-size categories are based on hospital beds and are specific to the hospital’s location and teaching status. More detailed information on the specific dollar amounts in each category of median household income and the number of hospital beds in each category can be found in the Nationwide Inpatient Sample description of data elements.^13^

^b^ Polysubstance use was defined as one or a combination of tobacco abuse, alcohol use disorder, or any other substance abuse.

^c^ Hypertensive disorder of pregnancy was defined as pregnancy-induced hypertension, gestational hypertension, or preeclampsia or eclampsia diagnosis during the index hospitalization.

The mean (SD) age of patients with PPCM and CS was 31 (9) years, and this was similar across all racial and ethnic groups (Table 1). Differences in the comorbidities, primary payer, median household income, hospital teaching status, location, bed size, and region are shown in Table 1. Notably, 519 Black patients (54.8%) and 119 Hispanic patients (50.4%) had Medicaid insurance, compared with 351 White patients (46.1%), and 20 Black patients (2.1%) and 15 Hispanic patients (6.4%) were uninsured, compared with 10 White patients (1.3%). Also, more Black patients were in the lowest quartile of median household income (467 patients [49.3%]), followed by Hispanic patients (64 patients [27.1%]). Black and Hispanic patients had higher prevalence of CKD, diabetes, and obesity compared with White patients. Hypertensive disorders of pregnancy were more common in Hispanic and Black patients compared with White patients (Table 1). APH occurred more frequently among Hispanic patients compared with Black and White patients. Black patients had the highest prevalence of chronic hypertension and hyperlipidemia, while polysubstance use was most common among White patients (Table 1). Clinical characteristics and outcomes of American Indian and Asian or Pacific Islander patients are in eTable 2 in the Supplement.

### Incidence of CS and Temporal Trends

**Figure 2.**
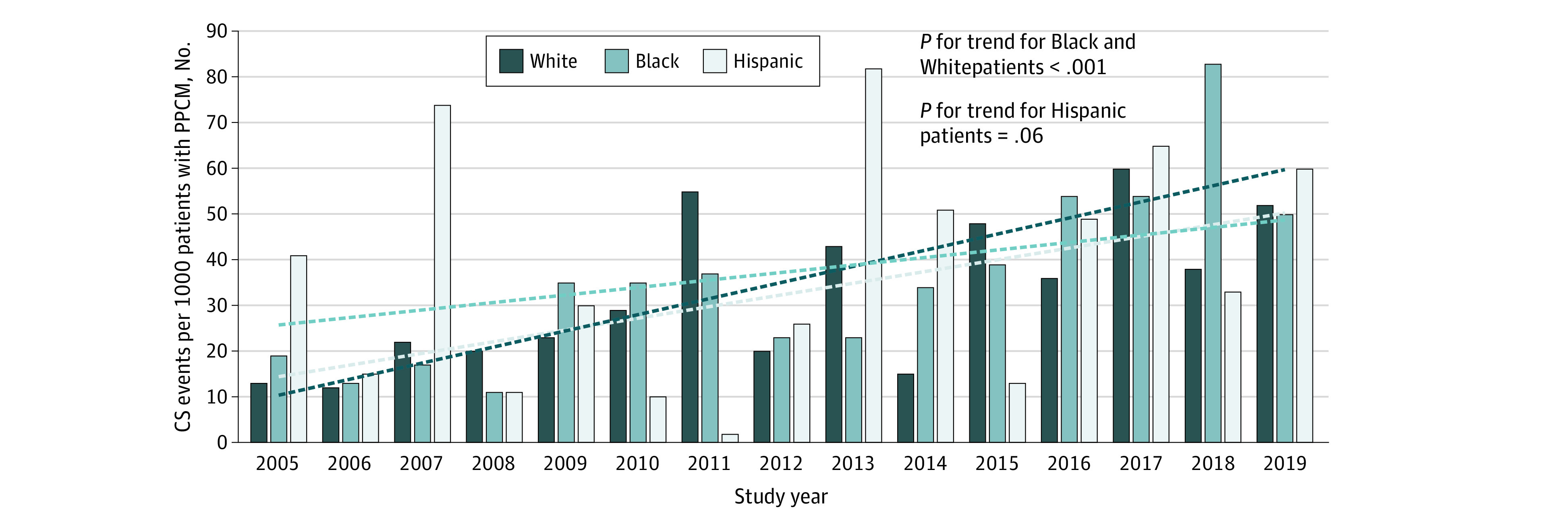
Temporal Trends in the Incidence Rates of Cardiogenic Shock (CS) Among Patients With Peripartum Cardiomyopathy (PPCM) Across the 3 Racial and Ethnic Groups, 2005-2019 Trend lines represent change in CS incidence rates.

The overall CS incidence rate was 35 events per 1000 patients with PPCM in the study cohort (Figure 2; eTable 3 in the Supplement). Stratified by race, Black and Hispanic patients had similar incidence rates (39 events per 1000 patients with PPCM), which were significantly higher compared with the incidence of 33 events per 1000 patients with PPCM among White patients (*P* < .001). The CS incidence rates increased across all races and ethnicities over the study period (Black patients: 19 events vs 50 events per 1000 patients with PPCM; Hispanic patients: 41 events vs 60 events per 1000 patients with PPCM; White patients, 13 events vs 52 events per 1000 patients with PPCM; *P* for trend < .001) (Figure 2). This upward trend was most consistent among Black patients, while Hispanic and White patients had more temporal year-to-year variabilities. On adjusted temporal trends evaluating the odds of CS in each study year compared with 2005, the variabilities in CS incidence among Hispanic and White patients were mostly not significant (eTable 4 in the Supplement). Overall, the odds of developing CS were higher in Black patients (aOR, 1.17 [95% CI, 1.15-1.57]; *P* < .001) and Hispanic patients (aOR, 1.37 [95% CI, 1.17-1.59]; *P* < 001) compared with White patients during the study period.

### Primary Outcome

Hispanic patients had the highest in-hospital mortality (49 patients [20.8%]), followed by Black patients (169 patients [17.8%]), then White patients (77 patients [10.1%]) (Table 2). In adjusted models, risk of in-hospital mortality was significantly higher among Black patients (aOR, 1.67 [95% CI, 1.21-2.32]; *P* = .002) and Hispanic patients (aOR, 2.20, 95% CI, 1.45-3.33]; *P* < .001) compared with White patients (Table 2).

**Table 2.** Clinical Outcomes of Hospitalized Patients With Peripartum Cardiomyopathy and Cardiogenic Shock Stratified by Race and Ethnicity

Outcomes	Patients, No. %			*P* value	Black^a^		Hispanic^a^	
	Black (n = 947)	Hispanic (n = 236)	White (n = 702)		aOR (95% CI)	*P* value	aOR (95% CI)	*P* value
In-hospital mortality	169 (17.8)	49 (20.8)	77 (10.1)	<.001	1.67 (1.21-2.32)	.02	2.20 (1.45-3.33)	<.001
Mechanical circulatory support use	199 (21.0)	84 (35.6)	159 (20.9)	<.001	1.09 (0.86-1.40	.45	2.23 (1.60-3.09)	<.001
ECMO	50 (5.3)	25 (10.6)	50 (6.6)	<.001	0.62 (0.41-1.01	.05	1.62 (0.95-2.71	.07
IABP	114 (12.0)	49 (20.8)	114 (15)	.008	0.93 (0.69-1.25	.60	1.65 (1.11-2.44)	.01
Temporal VAD	64 (6.8)	25 (10.6)	24 (3.2)	<.001	2.69 (1.63-4.42)	<.001	4.45 (2.45-8.08)	<.001
Heart transplant	59 (6.2)	5 (2.1)	60 (7.9)	.01	0.51 (0.33-0.78)	.02	0.15 (0.06-0.42)	<.001

Abbreviations: aOR, adjusted odds ratio; ECMO, extracorporeal membrane oxygenation; IABP, intraaortic balloon pump; VAD, ventricular assist device.

^a^ Compared with White patients and adjusted for age, primary payer, household income, hospital location region, teaching status, and comorbidities.

### Secondary Outcomes

Hispanic patients had the highest MCS device utilization rate (84 patients [35.6%]), followed by Black patients (199 patients [21.0%]) in crude analysis (Table 2). In the adjusted model, Hispanic patients were more likely to receive any MCS device (aOR, 2.23 [95% CI, 1.60-3.09]; *P* < .001), IABP (aOR, 1.65 [95% CI, 1.11-2.44]; *P* < .001), and VAD (aOR, 4.45 [95% CI, 2.45-8.08]; *P* < .001) compared with White patients. Black patients had higher odds of VAD use (aOR, 2.69 [95% CI, 1.63-4.42]; *P* < .001) compared with White patients (Table 2). IABP and ECMO use were comparable between Black and White patients. Notably, Black patients (aOR, 0.51 [95% CI, 0.33-0.78]; *P* = .02) and Hispanic patients (aOR, 0.15 [95% CI, 0.06-0.42]; *P* < .001) were significantly less likely to receive HT compared with White patients.

### In-Hospital Mortality Among Patients With PPCM and CS

**Figure 3.**
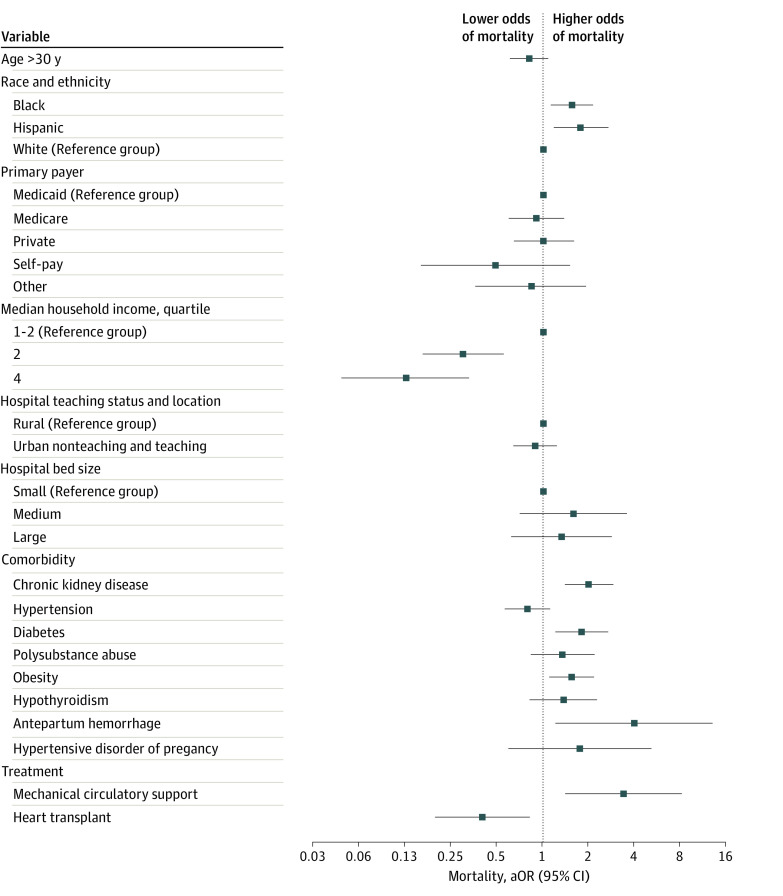
Multivariate Logistic Regression Analysis of Variables Associated With In-Hospital Mortality Among Hospitalized Patients With Peripartum Cardiomyopathy and Cardiogenic Shock aOR indicates adjusted odds ratio.

Besides race, other sociodemographic and clinical factors associated with increased mortality in patients with PPCM and CS were CKD (aOR, 2.00 [95% CI, 1.41-2.88]; *P* < .001), diabetes (aOR, 1.80 [95% CI, 1.21-2.69]; *P* < .001), obesity (aOR, 1.55 [95% CI, 1.10-2.16]; *P* < .001), APH (aOR, 3.97 [95% CI, 1.20-13.11]; *P* < .001), and MCS use (aOR, 3.38 [95% CI, 1.40-8.13]; *P* = .007). Higher median household income in the 51st to 75th (aOR, 0.30 [95% CI, 0.16-0.55]; *P* < .001) and 76th to 100th (aOR, 0.13 [95% CI, 0.05-0.33]) quartiles and HT (aOR, 0.40; 95% CI, 0.20-0.82]; *P* = .01) were associated with lower mortality (Figure 3).

### Subgroup Analysis

In subgroup analyses, we found a significantly higher adjusted risk of in-hospital mortality in Black and Hispanic patients compared with White patients among group of patients with CKD, obesity, APH, hypertensive disorders of pregnancy, and MCS use. Medicare and private insurance types were associated with lower odds of mortality (eFigure in the Supplement).

## Discussion

In this cohort study evaluating racial and ethnic disparities in the outcomes of hospitalized patients with PPCM, Black and Hispanic patients were more likely to develop CS, with increased odds of in-hospital mortality, compared with White patients. Black and Hispanic patients had higher utilization of MCS devices (IABP and VAD in Hispanic patients and VAD in Black patients) but were less likely to receive HT, compared with White patients.

Our analysis demonstrated an overall CS incidence rate of 3.5% and a temporal increase in CS incidence over a 15-year period (2005-2019) among Black, Hispanic, and White patients with PPCM. A study by Kolte et al^2^ found a CS incidence of 2.6% with a similar upward trend in CS rates from 1.0% in 2004 to 4.0% in 2011. Notably in our study, more CS events were reported between 2015 and 2019 compared with previous years, which is likely owing to early CS recognition with the initiation of better shock clinical guidelines over the years and improved coding and documentation practices with transition to *ICD-10-CM* codes in 2015.^29,30,31^

Prior studies have shown that Black patients with PPCM are more likely to present with a left ventricular ejection fraction (LVEF) less than 30% and are less likely to recover to an LVEF of 50% or higher.^26,32^ Black patients may also develop a more severe form of cardiomyopathy and worsening PPCM progression due to genetic or environmental factors.^26^ These factors may explain the higher CS incidence among Black patients compared with White patients with PPCM in our study.

Interestingly, we found that CS incidence rates among Hispanic patients were similar to Black patients despite historically reported lower risk of PPCM among Hispanic patients.^28^ Hispanic patients also had the highest odds of CS incidence and mortality, which is in contrast to previous studies.^28,33^ Our findings are likely due to a combination of multiple factors. We noted a higher prevalence of traditional cardiovascular disease risk factors, such as CKD, diabetes, and obesity, in Black and Hispanic patients compared with White patients. Additionally, Hispanic patients had the highest prevalence of hypertensive disorders of pregnancy and APH, which were independently associated with mortality in our study and are known to contribute to poor pregnancy outcomes.^34,35,36^ Given the novelty of the observed findings, prospective studies and randomized clinical trials are needed for further understanding.

Overall, the significantly higher mortality risk in Black and Hispanic patients compared with White patients is consistent with demonstrated racial and ethnic disparities in cardiovascular outcomes among pregnant and postpartum individuals with and without PPCM.^26,28^ We found higher mortality odds in the subgroups of Black and Hispanic patients with lower median income, CKD, obesity, and diabetes, which further substantiate the positive correlation between low socioeconomic status and higher cardiovascular comorbidity burden and poorer outcomes, including mortality in patients with cardiogenic shock.^37,38,39^ Similar factors were independently associated with increased mortality in the overall study cohort of patients with PPCM and cardiogenic shock.

MCS devices are used in CS with severe hemodynamic compromise refractory to medical therapy, sometimes as a bridge to a durable VAD or HT.^40,41^ The evidence on the association of MCS use compared with medical therapy with mortality in PPCM is variable and limited by small observational studies, case series, and reports.^42,43,44,45,46,47,48^ We found worse mortality outcomes in Black and Hispanic patients compared with White patients, despite relatively higher MCS utilization rates. This paradox may be explained by more severe disease refractory to the benefit of circulatory support devices among Black and Hispanic patients with PPCM and CS. Early application of MCS has been associated with improved mortality rates, and Black patients are often diagnosed with PPCM late postpartum, with significantly low LVEF at the time of presentation, which may impact the clinical effectiveness of MCS devices.^26,47^

It is also possible that White patients were more successfully bridged to HT owing to less severe CS, accounting for their observed lower MCS utilization, higher HT rates, and lower mortality odds. It is difficult to validate these individual hypotheses owing to lack of granular clinical information in the NIS, like vital signs, laboratory findings, imaging, and echocardiography, to objectively evaluate disease severity. There are currently no clear clinical guidelines on the use of MCS specifically in PPCM complicated by CS, which may lead to discrepancies in the use of these therapeutic approaches across racial and ethnic groups. Larger prospective studies and clinical trials are required to provide evidence-based recommendations on MCS use in this population.

A study by Bouabdallaoui et al^49^ reported that HT was associated with improved survival rates in PPCM patients who did not respond favorably to medical therapy, which is consistent with our findings. Although Black and Hispanic patients were less likely to receive HT compared with White patients, we did not find a significant association between race or ethnicity and mortality among the subgroup of patients who received HT. We are unable to ascertain patient eligibility, selection criteria, and contraindications that may explain the racial and ethnic disparities noted in HT in this study owing to limitations of the NIS database. Nonetheless, significant racial and ethnic differences have been reported in HT utilization in the US. In a study of adult heart recipients from 2011 to 2020 in the United Network for Organ Sharing database,^50^ Black and Hispanic patients were less likely to undergo HT despite an increase in the proportion listed for transplantation.

### Limitations

Our study has limitations, largely intrinsic to retrospective studies using administrative data, such as the HCUP-NIS. Our analysis relied on *ICD-9-CM* and *ICD-10-CM* codes,^14,15^ which are subject to coding errors. Although we excluded patients with previous diagnoses of heart failure or structural heart diseases that could explain pregnancy-related cardiac dysfunction, it is still challenging for clinicians to determine whether a patient had unrecognized underlying familial cardiomyopathy unmasked by pregnancy.^51^ We recognize the possibility that some patients were pregnant more than once, with multiple PPCM hospitalizations. However, this number is likely to be very low, with minimal impact on the PPCM with CS incidence rates because health care practitioners often discourage subsequent pregnancies in patients with PPCM owing to high recurrence and mortality risk.^52,53^ Despite these limitations, our study is derived from a large sample size of nationally representation inpatient data with generalizable results to the US population.

## Conclusions

This cohort study found racial and ethnic disparities in the incidence and outcomes of cardiogenic shock complicating PPCM. Black and Hispanic patients were more likely to experience in-hospital mortality despite higher MCS use. Black and Hispanic patients were less likely to receive HT. These findings prompt the need for more research to better understand factors contributing to the observed racial and ethnic disparities, which will be essential in guiding interventions targeted at improving outcomes for patients with PPCM.

## Notes

Supplement. **eTable 1.** Clinical Variables and *ICD-9* and *ICD-10* Diagnosis and Procedure Codes **eTable 2.** Baseline Characteristics of American Indian and Asian or Pacific Islander Patients **eTable 3.** Incidence Rates of Cardiogenic Shock Among Hospitalized Patients With Peripartum Cardiomyopathy in the US, 2005-2019 **eTable 4.** Temporal Trends in the Adjusted Odds of Cardiogenic Shock Among Patients With Peripartum Cardiomyopathy from 2005-2019, Stratified by Race and Ethnicity **eFigure.** Factors Associated With In-Hospital Mortality Among Subgroups of Patients With Peripartum Cardiomyopathy and Cardiogenic Shock
