# Supplementary material for: Racial and Ethnic Disparities in the Trends and Outcomes of Cardiogenic Shock Complicating Peripartum Cardiomyopathy
Source: JAMA Netw Open. 2022 Jul 5;5(7):e2220937. doi: 10.1001/jamanetworkopen.2022.20937 (PMC9257562; doi:10.1001/jamanetworkopen.2022.20937)
Supplement: Supplement. — eTable 1. Clinical Variables and ICD-9 and ICD-10 Diagnosis and Procedure Codes eTable 2. Baseline Characteristics of American Indian and Asian or Pacific Islander Patients eTable 3. Incidence Rates of Cardiogenic Shock Among Hospitalized Patients With Peripartum Cardiomyopathy in the US, 2005-2019 eTable 4. Temporal Trends in the Adjusted Odds of Cardiogenic Shock Among Patients With Peripartum Cardiomyopathy from 2005-2019, Stratified by Race and Ethnicity eFigure. Factors Associated With In-Hospital Mortality Among Subgroups of Patients With Peripartum Cardiomyopathy and Cardiogenic Shock [file jamanetwopen-e2220937-s001.pdf]

Supplemental Online Content

Olanipekun T, Abe T, Effoe V, et al. Racial and ethnic disparities in the trends and outcomes of cardiogenic shock complicating peripartum cardiomyopathy. *JAMA Netw Open.* 2022;5(7):e2220937. doi:10.1001/jamanetworkopen.2022.20937

eTable 1. Clinical Variables and *ICD-9* and *ICD-10* Diagnosis and Procedure Codes

eTable 2. Baseline Characteristics of American Indian and Asian or Pacific Islander Patients

eTable 3. Incidence Rates of Cardiogenic Shock Among Hospitalized Patients With Peripartum Cardiomyopathy in the US, 2005-2019

eTable 4. Temporal Trends in the Adjusted Odds of Cardiogenic Shock Among Patients With Peripartum Cardiomyopathy from 2005-2019, Stratified by Race and Ethnicity

eFigure. Factors Associated With In-Hospital Mortality Among Subgroups of Patients With Peripartum Cardiomyopathy and Cardiogenic Shock

This supplemental material has been provided by the authors to give readers additional information about their work.

© 2022 Olanipekun T et al. *JAMA Network Open.*

eTable 1. Clinical Variables and *ICD-9* and *ICD-10* Diagnosis and Procedure Codes

Variables	CM/CCS/PR codes	ICD-10-CM/CCS/PR codes
**Comorbidities and Clinical characteristics**		
Pre-eclampsia	642.40, 642.41, 642.42, 642.43, 642.44.<br>642.50, 642.51, 642.52, 642.53, 642.54.	O14.0, O14.1, O14.2, O14.9.<br><br>O14.10, O14.12, O14.13.
Eclampsia	642.60, 642.61, 642.62, 642.63, 642.64	O15.0, O15.1, O15.2, O15.9
Pregnancy associated HTN	642.30, 642.31, 642.32, 642.33, 642.34	O13.1, O13.2, O13.3, O13.9
Tobacco use disorder	305.1	F17203, F17208, F17209, F17210, F17211, F17213, F17218, F17219, F17220, F17221, F17223, F17228, F17290, F17291, F17293, F17298, F17299
Hypothyroidism	244.9	E039
Chronic kidney disease	585.1 – 585.5	N181, N182, N183, N184, N185, N189
Obesity	278.0 – 278.03	E66.01 - 66.2, E66.8-9, Z68.31 Z68.4
Hypertension	401- 405	I10
Diabetes uncomplicated	250.00 – 250.03, 250.7	E11.9
Diabetes Complicated	250.4 – 250.6	E11.0 – E11.8
Hyperlipidemia	272.4	E78.0 – E78.5
Hemodialysis	39.95	N18.6, Z99.2, Z49.31, Z49.01
Chronic Liver disease	070.22, 070.23, 070.32, 070.33, 070.44, 070.54, 456.0, 456.1, 456.20, 571.0, 571.2-571.9, 572.3, 572.8, V42.7	K70.0, - K70.4, K70.9, K71.3 - K71.9, K73.0 - K73.9, K74.0 - K74.6, K76.0 - K76.9
Aspirin use	V58.66	Z79.82, Z79.02
**Outcomes**		
Mechanical Circulatory Support	37.61, 39.65, 37.68	5A02110, 5A02210, 5A15223, 5A0211D, 5A0221D, 02HA3RJ, O2HA4RJ
Intra-aortic balloon pump	37.61	5A02110, 5A02210
Extracorporeal membrane oxygenation	39.65	5A15223
Temporary Ventricular assist device	37.68	5A0211D, 5A0221D, 02HA3RJ, O2HA4RJ
Heart Transplant	37.51	02YA0Z0, 02YA0Z1, 02YA0Z2

© 2022 Olanipekun T et al. *JAMA Network Open.*

eTable 2. Baseline Characteristics of American Indian and Asian or Pacific Islander Patients

Variables	Asians/Pacific (N=59)	Native Americans (N=10)
Age (years), mean (SD)	27 (10)	32 (9)
**Co-morbidities**		
Hypothyroidism	7.1	5.7
Obesity	8.5	0
Diabetes mellitus	8.5	0
Chronic kidney disease	16.9	0
Hypertension	16.9	0
Polysubstance abuse[a]	2.1	0
Hyperlipidemia	16.9	0
Hypertensive disorders of pregnancy[b]	4.2	8.5
Antepartum hemorrhage	4.0	8.8
**Outcomes**		
In-hospital mortality	15.3	50
Mechanical circulatory support use	25	0
ECMO	0	0
IABP	8.5	0
Temporal VAD	16.7	0
Heart transplant	0	0

Values are expressed as percentages and arranged in columns unless otherwise indicated
[a] Polysubstance use was defined as one or combination of tobacco abuse, alcohol use disorder or any other substance abuse
[b] Hypertensive disorder of pregnancy was defined as one of pregnancy induced hypertension, gestational hypertension, or preeclampsia – eclampsia diagnosis during the index hospitalization
Abbreviations: SD – standard deviation.

© 2022 Olanipekun T et al. *JAMA Network Open.*

eTable 3. Incidence Rates of Cardiogenic Shock Among Hospitalized Patients With Peripartum Cardiomyopathy in the US, 2005-2019

**Study Year**	**White women**	**Black women**	**Hispanic women**
2005	13	19	41
2006	12	13	15
2007	22	17	74
2008	20	11	11
2009	23	35	30
2010	29	35	10
2011	55	37	0
2012	20	23	26
2013	43	23	82
2014	15	34	51
2015	48	39	13
2016	36	54	49
2017	60	54	65
2018	38	83	33
2019	52	50	60

Incidence rate is expressed as the number of cardiogenic shock cases per 1000 hospitalized women with Peripartum cardiomyopathy

© 2022 Olanipekun T et al. *JAMA Network Open.*

eTable 4. Temporal Trends in the Adjusted Odds of Cardiogenic Shock Among Patients With Peripartum Cardiomyopathy from 2005-2019, Stratified by Race and Ethnicity

	**Study year**	**P-value**	**aOR**	**95% CI**	
**Black women**	2005	Reference			
	2006	0.34	1.49	0.66	3.35
	2007	0.01	2.66	1.29	5.50
	2008	0.03	2.23	1.08	4.61
	2009	0.02	2.35	1.16	4.77
	2010	0.01	3.09	1.56	6.12
	2011	<0.001	6.67	3.45	12.90
	2012	0.41	1.38	0.64	2.96
	2013	<0.001	4.64	2.37	9.10
	2014	0.18	1.66	0.79	3.47
	2015	<0.001	5.52	2.83	10.76
	2016	0.01	3.22	1.63	6.36
	2017	<0.001	5.11	2.64	9.89
	2018	0.01	3.22	1.63	6.35
	2019	<0.001	4.82	2.48	9.37
**Hispanic women**	**Study year**	**P-value**	**aOR**	**95% CI**	
	2005	Reference			
	2006	0.13	0.55	0.26	1.19
	2007	0.32	0.71	0.36	1.40
	2008	0.02	0.42	0.20	0.87
	2009	0.93	0.97	0.54	1.75
	2010	0.39	1.29	0.73	2.28
	2011	0.73	1.10	0.63	1.94
	2012	0.13	0.62	0.33	1.15
	2013	0.50	0.81	0.44	1.49
	2014	0.27	1.38	0.78	2.46
	2015	0.24	1.40	0.79	2.48
	2016	0.02	1.93	1.10	3.36
	2017	0.02	1.94	1.11	3.38
	2018	<0.001	2.88	1.69	4.93
	2019	0.08	1.63	0.94	2.82
**White women**	**Study year**	**P-value**	**aOR**	**95% CI**	
	2005	Reference			
	2006	0.82	1.07	0.62	1.84
	2007	0.01	0.38	0.18	0.81
	2008	0.55	0.83	0.46	1.52
	2009	0.86	0.95	0.54	1.69

© 2022 Olanipekun T et al. *JAMA Network Open.*

	2010	0.22	1.47	0.79	2.76
	2011	0.39	0.71	0.32	1.57
	2012	0.03	2.19	1.09	4.43
	2013	0.35	0.62	0.23	1.68
	2014	0.66	0.80	0.29	2.19
	2015	0.15	1.67	0.84	3.33
	2016	<0.001	3.85	1.90	7.81
	2017	0.30	1.52	0.69	3.37
	2018	0.02	2.68	1.19	6.04
	2019	<0.001	8.62	4.46	16.66

aOR- Adjusted Odd ratio
CI – Confidence interval

© 2022 Olanipekun T et al. *JAMA Network Open.*

eFigure. Factors Associated With In-Hospital Mortality Among Subgroups of Patients With Peripartum Cardiomyopathy and Cardiogenic Shock

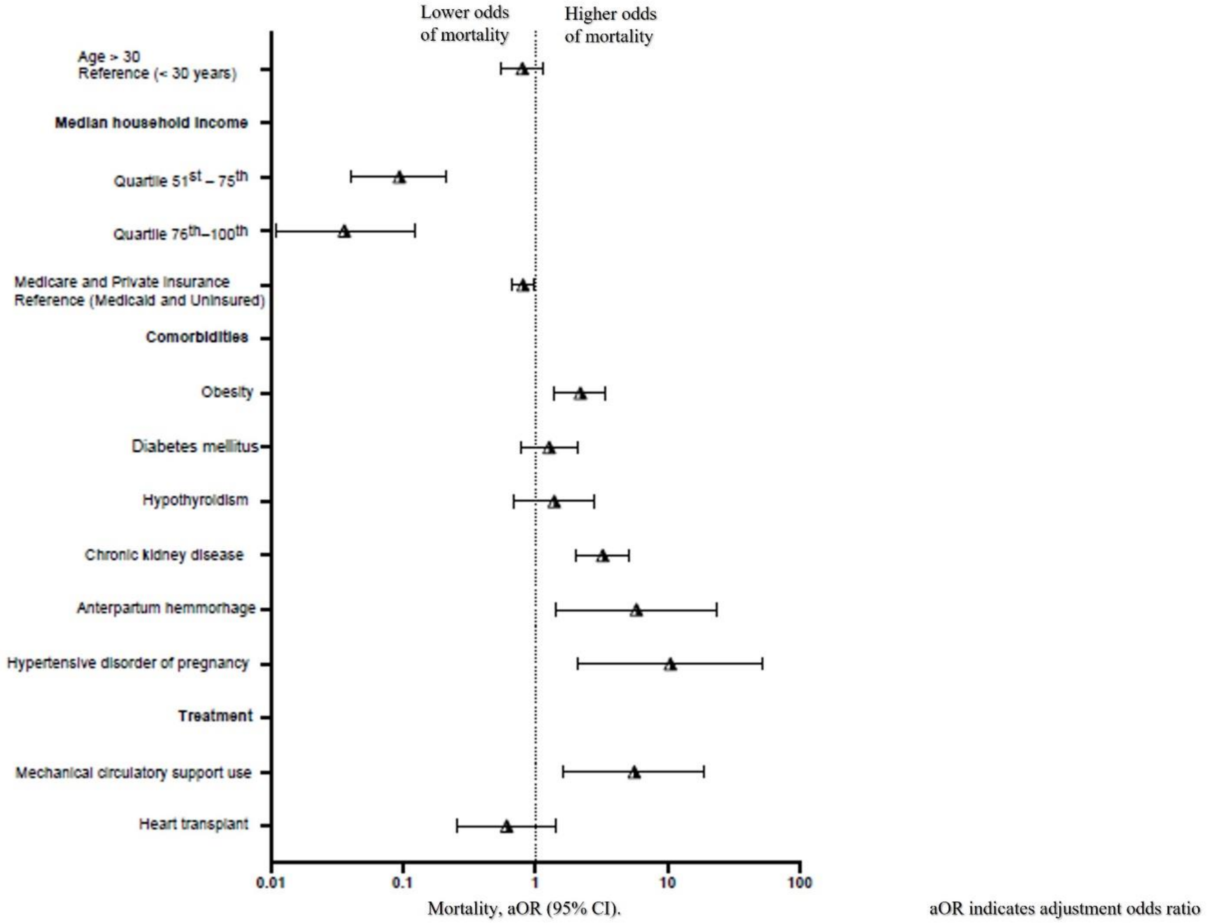

© 2022 Olanipekun T et al. *JAMA Network Open.*
